# Improvement of Imiquimod Solubilization and Skin Retention via TPGS Micelles: Exploiting the Co-Solubilizing Effect of Oleic Acid

**DOI:** 10.3390/pharmaceutics13091476

**Published:** 2021-09-15

**Authors:** Martina Ghezzi, Silvia Pescina, Andrea Delledonne, Ilaria Ferraboschi, Cristina Sissa, Francesca Terenziani, Paula De Freitas Rosa Remiro, Patrizia Santi, Sara Nicoli

**Affiliations:** 1 Department of Food and Drug, University of Parma, Parco Area delle Scienze 27/A, 43124 Parma, Italy; martina.ghezzi@unipr.it (M.G.); silvia.pescina@unipr.it (S.P.); patrizia.santi@unipr.it (P.S.); pdefreitasrosa@gmail.com (P.D.F.R.R.); 2 Department of Chemistry, Life Science and Environmental Sustainability, University of Parma, Parco Area delle Scienze 17/A, 43124 Parma, Italy; andrea.delledonne@unipr.it (A.D.); ilaria.ferraboschi@studenti.unipr.it (I.F.); cristina.sissa@unipr.it (C.S.); francesca.terenziani@unipr.it (F.T.); 3 Department of Engineering of Materials and of Bioprocesses, School of Chemical Engineering, University of Campinas (UNICAMP), Av. Albert Einstein 500–Cidade Universitaria, Campinas 13083-852, Brazil

**Keywords:** imiquimod, polymeric micelles, skin delivery, fatty acids, solubility, skin cancer

## Abstract

Imiquimod (IMQ) is an immunostimulant drug approved for the topical treatment of actinic keratosis, external genital-perianal warts as well as superficial basal cell carcinoma that is used off-label for the treatment of different forms of skin cancers, including some malignant melanocytic proliferations such as lentigo maligna, atypical nevi and other in situ melanoma-related diseases. Imiquimod skin delivery has proven to be a real challenge due to its very low water-solubility and reduced skin penetration capacity. The aim of the work was to improve the drug solubility and skin retention using micelles of d-α-tocopheryl polyethylene glycol 1000 succinate (TPGS), a water-soluble derivative of vitamin E, co-encapsulating various lipophilic compounds with the potential ability to improve imiquimod affinity for the micellar core, and thus its loading into the nanocarrier. The formulations were characterized in terms of particle size, zeta potential and stability over time and micelles performance on the skin was evaluated through the quantification of imiquimod retention in the skin layers and the visualization of a micelle-loaded fluorescent dye by two-photon microscopy. The results showed that imiquimod solubility strictly depends on the nature and concentration of the co-encapsulated compounds. The micellar formulation based on TPGS and oleic acid was identified as the most interesting in terms of both drug solubility (which was increased from few µg/mL to 1154.01 ± 112.78 µg/mL) and micellar stability (which was evaluated up to 6 months from micelles preparation). The delivery efficiency after the application of this formulation alone or incorporated in hydrogels showed to be 42- and 25-folds higher than the one of the commercial creams.

## 1. Introduction

Actinic keratosis (AK) is a precancerous lesion caused by excessive exposure to light whose appearance is related to several mechanisms such as oxidative stress, immunosuppression, inflammation, altered proliferation and dysregulation of cell growth, impaired apoptosis, mutagenesis and papilloma virus (HPV) [1]. As actinic keratosis is the most common precursor of cutaneous invasive squamous cell carcinomas, its treatment appears to be imperative. In this sense, imiquimod (IMQ), a synthetic drug belonging to the class of imidazoquinolones, has widely demonstrated its usefulness thanks to its immunostimulant activity mediated by the binding to the toll-like receptors 7 and 8. These receptors play a pivotal role in controlling innate immune system response as they induce the secretion of pro-inflammatory cytokines such as interferons (IFN-α, IFN-γ), tumor necrosis factor-alpha (TNF-α) and interleukin 12 (IL-12) [2,3]. Moreover, imiquimod topical application promotes Langerhans cells stimulation with subsequential migration to local lymph nodes resulting in the adaptive immune system activation [4,5]. Together with imiquimod indication for the treatment of actinic keratosis, other uses of the drug have been approved by the U.S. Food and Drug Administration (FDA) including the treatment of external genital and perianal warts and superficial basal cell carcinoma [6]. Furthermore, an increasing interest for imiquimod potential in dermatological field has been registered as a consequence of its several off-labels indications towards skin infections and skin cancers such as Bowen’s disease [7], nodular basal cell carcinoma (nBCC) [8,9], lentigo maligna, atypical nevi and other in situ melanoma-related diseases [10,11,12]. The data on malignant melanocytic proliferations, although preliminary, highlighted that, even if surgical excision remains the preferred approach, the use of imiquimod can represent a valid alternative in specific cases such as the management of persistently positive melanoma margins and the treatment of large and less defined lesions on the head and neck, most of the time requiring a disfiguring surgical intervention. Finally, imiquimod topical use as adjuvant for cancer vaccines is presently under investigation and, at the moment, it shows good tolerability and a systemic immune response [13,14].

Despite imiquimod proven effectiveness for skin disorders treatment, its topical administration has demonstrated to be quite challenging due to the very low water-solubility and the limited penetration capacity [15], presumably related to imiquimod very low affinity for stratum corneum (SC) and underlying tissues. A successful strategy to overcome these issues could be found in the use of micelles, self-assembling colloidal systems composed of a hydrophobic core, playing an essential role in improving the solubility of poorly water-soluble compounds, surrounded by an external hydrophilic corona. Polymeric micelles have demonstrated the ability to facilitate drugs penetration into the skin by several pathways such as the transcellular route [16], the intercluster penetration and the follicular way, thus promoting a slow and sustained drug release by creating drug depots in the tissue [17]. Furthermore, considering the amphiphilic structure of the polymers, an augment of drug retention in the skin could be favored by their capacity to alter the spatial structure of lipids and keratin in the SC reducing its resistance to drug penetration [18,19], in addition to a modification of barrier lipophilicity [20]. Among the polymers used for micelles preparation, tocopheryl polyethylene glycol 1000 succinate (TPGS), a water-soluble derivative of vitamin E with high biocompatibility and biodegradability, has been widely appreciated in several fields of drug delivery [21,22,23] because of solubilization capacity and permeation enhancing property [24].

The aim of this work was to optimize imiquimod solubility and skin retention by using TPGS-based micelles. In order to further increase imiquimod solubility, lipophilic compounds that could, at least in principle, enter the micellar core and enhance the affinity for the drug, will be co-encapsulated. This kind of strategy has already been proposed for increasing drug loading in other nanocarriers such as niosomes [25] and lipid-based particles [26,27] but, at our knowledge, it has never been investigated in case of micelles. In particular, the research included (1) the determination of imiquimod solubility in TPGS micelles, with or without co-solubilizing agents, primarily represented by fatty acids; (2) the characterization of the micelles in terms of size and stability; (3) the selection of an appropriate thickening agent to transform the micellar formulation into a gel suitable for skin application; (4) the evaluation of micelles performance on the skin through the quantification of imiquimod retention in the skin layers and the visualization of a micelle-loaded fluorescent dye by two-photon microscopy.

## 2. Materials and Methods

### 2.1. Materials

Imiquimod (IMQ; 1-isobutyl-1H-imidazo[4,5-c]quinolin-4-amine; MW 240.3 g/mol; pKa 7.3; water solubility 0.6–2.4 µg/mL) was purchased from Hangzhou Dayangchem (Zhejiang, China) while tocopheryl polyethylene glycol 1000 succinate (TPGS) was gifted from PMC ISOCHEM (Vert-Le-Petit, France). Oleic acid was purchased from Alfa Aesar (Karlsruhe, Germany) whereas isostearic acid was a kind gift from Biochim (Milan, Italy). Linoleic acid and linolenic acid were purchased respectively from Alfa Aesar (Karlsruhe, Germany) and Sigma-Aldrich (St. Louis, MO, USA). Plurol^®^ Oleique (mixture of polyglyceryl-3 esters of oleic acid with a predominant diester fraction) and Peceol™ type 40 were a kind gift from Gattefossé (Saint-Priest, France). Span^®^ 80 was purchased from Bregaglio (Biassono, Italy). Natrosol™ 250 M (MW 720 kDa) was a kind gift of Ashland (Wilmington, DE, USA) whereas xanthan gum was purchased from Sigma-Aldrich (Saint Louis, MO, USA). Sodium carboxymethylcellulose E466 (MW 25 kDa) was purchased from A.C.E.F. S.p.A. (Fiorenzuola d’Arda, Italy) whereas Carbopol^®^ 934NF (MW 300 kDa) was purchased from Lubrizol (Wickliffe, OH, USA). Sodium hyaluronate (HA; MW 1000 kDa) was a kind gift from IBSA Farmaceutici Srl (Lodi, Italy) while PVA Gohsenol™ EG-30P (MW 98 kDa) was purchased from Nippon Synthetic Chemical Industry Co. Ltd. (Osaka, Japan). Albumin from bovine serum and nile red (NR; 9-(diethylamino)-5H-benzo[a]phenoxazin-5-one; MW 318.4 g/mol) were purchased from Sigma Aldrich (St. Louis, MO, USA). High purity water was used (Arium^®^ comfort, Sartorius, Goettingen, Germany); all solvents were of analytical grade.

### 2.2. Imiquimod Quantification Method

IMQ quantification was performed by HPLC (Flexar, Perkin Elmer, Waltham, MA, USA) equipped with a reverse-phase C_18_ column (Kinetex C18 2.6 μm, 100 Å, 75 × 4.6 mm, Phenomenex, Torrance, CA, USA) and a C_18_ guard column (SecurityGuard Widepore C18, Phenomenex, Torrance, CA, USA). The mobile phase, composed of methanol:acetonitrile:water:triethylamine (180:270:530:20), was pumped at a flow rate of 0.5 mL/min. In these conditions, the retention time of the drug was about 4 min. Imiquimod detection was carried out through UV or fluorescence detection. In particular, imiquimod solubility was mostly assessed by UV absorbance (λ 242 nm; injection volume: 10 μL), whereas fluorescence measurement (λ_exc_ 260 nm, λ_em_ 340 nm, injection volume: 1 μL) was used in order to evaluate imiquimod accumulation and permeation through tissues. Calibration curves in the intervals 0.5–15 µg/mL for UV and 0.05–2.5 µg/mL for fluorescence were built starting from a stock solution prepared by dissolving approximately 2 mg of imiquimod in 20 mL of 0.1 M HCl. The HPLC methods were previously validated for sensitivity, precision and accuracy [28].

### 2.3. Fatty Acids Quantification Method

Fatty acids quantification was performed by HPLC (Flexar, Perkin Elmer, Waltham, MA, USA) equipped with a reverse-phase C_18_ column (Nova-Pack C18, 150 × 3.9 mm, Waters, Milford, MA, USA) for oleic acid and with a reverse-phase C_8_ column (Aeris WIDEPORE XB, 150 × 4.60 mm, Phenomenex, Torrance, CA, USA) for linoleic and linolenic acid. In both cases, the columns were thermostated at 65 °C and the flow rate was 1.6 mL/min. For oleic acid, the injection volume was 50 µL and the mobile phase was composed of acetonitrile:water with trifluoroacetic acid (TFA) 0.1% in ratio 65:35 (*v*/*v*), whereas linoleic and linolenic acids quantification was carried out by using acetonitrile:water with TFA 0.1% in ratio 60:40 (*v*/*v*) and an injection volume of 100 µL. Absorbance of oleic acid, whose retention time in these conditions was about 12 min, was measured at 210 nm. The same wavelength was used for quantifying both linoleic and linolenic acid, whose retention times were about 2.7 min and 2.1 min respectively. Details on calibration curves, RSD%, RE% and LOQ values are reported in Table S1.

### 2.4. Preparation of Blank Polymeric Micelles

#### 2.4.1. Method 1

TPGS micelles were prepared by direct dissolution of TPGS (conc. approximately 20, 40, 100, 200 mM) in high purity water to obtain formulations T20, T40, T100 and T200. TPGS micelles were then saturated with oleic acid (O), linoleic acid (L), linolenic acid (LN) and isostearic acid (I) or with oleic acid esters, namely Plurol^®^ Oleique (PO), Peceol™ (P) or Span 80^®^ (SP) (see Table 1 for details on the structures). Briefly, the micellar solutions were added with the above-mentioned excipients at 4:1 volumetric ratio and the obtained mixtures were subjected to 100 revolutions (made by hand) to ensure complete saturation of the aqueous phase. After saturation, the oily phase was removed and a 2-folds filtration (regenerated cellulose, Minisart RC 0.2 µm, Sartorius, Gottingen, Germany) of the aqueous phase was performed in order to get a clear micellar solution. The obtained formulations (composition and details are shown in Table 2) were then saturated with imiquimod (see Section 2.5).

#### 2.4.2. Method 2

In order to deepen the effect of different preparation methods on IMQ solubility, other processes for micelles development were tested using only TPGS and oleic acid. In this case, a mixture of methanol and oleic acid in ratio 1:1 was left under magnetic stirring until a clear solution was obtained. Then, 4 mL of this solution were injected into 8 mL of T20 and left under magnetic stirring for 30 min. Afterwards, methanol was evaporated by stirring the system in a water bath at 50 °C for approximately 2 h. Finally, the system was filtered 2 times (regenerated cellulose, Minisart RC 0.2 µm, Sartorius, Gottingen, Germany) to eliminate the excess of oily phase. Even after filtration, the solution remained opalescent (see DLS analysis, Table 3 for details). The obtained formulation (composition and details are shown in Table 2) was then saturated with imiquimod (see Section 2.5).

#### 2.4.3. Method 3

A total of 2 mL of oleic acid was saturated with imiquimod (approximately IMQ conc.: 74 mg/mL). After filtration, the saturated solution was added in a 1:4 ratio to T20, as in the method 1 (Section 2.4.1).

### 2.5. Imiquimod Loading in the Polymeric Micelles

In order to load (IMQ), an excess of the drug was added to the blank micellar formulations prepared (a list is reported in Table 2). Mixtures were left under magnetic stirring at room temperature for 48 h. Then, the suspension was centrifuged at 12,500 rpm for 15 min and the supernatant was sampled and analyzed by HPLC-UV or HPLC-fluorescence for imiquimod solubility determination, after 1:100 dilution in 0.01 M HCl.

### 2.6. Micelles Characterization and Stability

Size, polydispersity index (PDI) and surface charge of blank and IMQ-loaded micelles were measured by Dynamic Light Scattering (DLS) using Zetasizer Nano-ZSP (Malvern Instruments, Malvern, UK). Measurements were carried out at 25 °C after 10-folds samples dilution in high purity water. Surface charge determination was performed by 40-folds samples dilution in 0.5 mM KCl.

Stability over time (storage at room temperature) was also determined. Imiquimod concentration in TO20 and TI20 micelles was evaluated after 3 and 6 months from the preparation, using the HPLC method previously described and by diluting the samples 1:100 in 0.01 M HCl. pH of TO20 and TI20 micelles was measured after 0, 100 and 200 days from micelles preparation. In case of TO20 micelles, in order to further describe polymeric micelles behavior over time, size was measured after 100 days from samples preparation. Stability over time of TLN20 and TL20 was evaluated visually, as the formation of a white precipitate occurred after 14 days from micelles preparation.

### 2.7. Formulation of Water-Based and Micelles-Based Hydrogels

Different polymers were considered as thickening agents for the micellar solution. Polymers evaluated were 1% xanthan gum (XG), 2% hydroxyethyl cellulose (HEC), 1% sodium hyaluronate (HA), 4% sodium carboxymethylcellulose (CMC), 0.5% Carbopol^®^ 934 (CP) and 15% polyvinyl alcohol EG-30P (PVA). Details on the characteristics of the polymers used are reported in the material Section (Section 2.1).

#### Hydrogels Preparation

Hydrogels were prepared by hydration of the polymers in high purity water. In case of xanthan gum (XG), hydroxyethyl cellulose (HEC), sodium hyaluronate (HA) and Carbopol^®^ (CP), the polymer was added to high purity water and left under magnetic stirring at room temperature until complete hydration. Carbopol^®^ solution was then added with 2 M NaOH until the formation of the hydrogel was observed. Sodium carboxymethylcellulose (CMC) hydrogel was prepared by intermittent vortexing after dispersing the polymer in high purity water. Polyvinyl alcohol (PVA) gel formulation was obtained by dispersion of the polymer in water overnight at room temperature followed by heating at 90 °C until complete hydration. After hydration, all hydrogels were left to rest overnight. When necessary, in order to remove bubbles, hydrogels were centrifuged at 3000 rpm for 10 min. The same procedures described above were followed to prepare micelles-based hydrogels. In this case, hydrogels were obtained by hydration of the polymers in both the blank TO20 and IMQ-loaded TO20 micelles. pH of the formulations was measured for water-based as well as IMQ-loaded TO 20-based hydrogels.

### 2.8. Evaluation of Micelles Diffusion in Water-Based Hydrogels

A preliminary experiment was performed in order to describe the ability of TO20 micelles to diffuse across the different hydrogels. The experimental procedure was carried out as a preliminary screening to select the most promising hydrogels for further testing. With this aim, a saturated solution of nile red in blank TO20 micelles was prepared. Briefly, 10 µL of a 10 mg/mL nile red solution in DMSO (conc. 10 mg/mL) were added to 1.5 mL of blank TO20, which was then centrifuged at 13000 rpm for 5 min to precipitate the excess of nile red.

In a 3 mL plastic tube, 2.5 g of hydrogels were gently transferred, taking care to avoid bubbles formation. Next, 200 µL of nile red-loaded micelles were deposed on the surface of each hydrogel (Figure S1). Diffusion of the micelles through the hydrogels was visually evaluated at predetermined time points (1 h, 2 h, 24 h).

### 2.9. Skin Accumulation and Permeation Experiments

Skin accumulation and permeation experiments were performed by using porcine tissues obtained from a local slaughterhouse (Macello Annoni Spa, Madonna dei Prati, Busseto, Parma, Italy). The skin was excised from the outer part of pig ears (breed, Large White and Landrance; weight, 145–190 kg; age, 10–11 months; sex, male and female) within 3 h from animal death, and separated from the underlying cartilage utilizing a scalp. Afterwards, the tissue was frozen at −20 °C and used within 3 months. Defrosted skin was mounted on glass Franz-type diffusion cells (DISA, Milano, Italy; 0.6 cm^2^ surface area) with the stratum corneum facing the donor compartment. To guarantee sink conditions, the receptor compartment was filled with 1% *w/v* bovine serum albumin solution in PBS pH 7.4 (IMQ solubility: 143 ± 3 μg/mL) [37]. Formulations tested were TO20, TO20-2, TO100 (see Table 2 for composition), micellar-based hydrogels HA and XG, and the commercial formulation Imunocare^®^ (composition: isostearic acid, benzyl alcohol, cetyl alcohol, stearyl alcohol, white soft paraffin, polysorbate 60, sorbitan stearate, glycerol, methyl hydroxybenzoate, propyl hydroxybenzoate, xanthan gum and purified water; IMQ concentration: 50 mg/g) as control. All the donors were applied at infinite dose (200 mg/cm^2^, occluded) for 6 h. At the end of the experiment, the receptor solution was sampled, the formulation was removed from the donor compartment and the skin surface was rinsed with water to clear away the last traces of micelles. The tissue was then delicately dried and tape-stripped twice (Scotch Booktape #845, 3 M Co., St. Paul, MN, USA) to remove the most external layers of stratum corneum and eliminate any traces of imiquimod-loaded formulation, thus avoiding potential contamination. Consequently, skin samples were heated (hairdryer for 60 s) and epidermis was separated from dermis using a spatula. This separation procedure could slightly affect skin distribution, increasing drug levels in the deeper skin layers. Nonetheless, the short heat application time should limit this phenomenon; additionally, all tissues were treated equally, and any possible increase in drug diffusion to the dermis would be comparable for all samples. IMQ extraction from tissues was carried out by treating tissues with 1 mL solution of oleic acid:methanol (1:3) for the epidermis and 1 mL solution of PEG 400:methanol: 1 M HCl (1:2:2) for the dermis. Samples were left under these conditions overnight at room temperature, centrifuged at 12,500 rpm for 10 min and analyzed by HPLC-fluorescence. In order to evaluate IMQ permeation, 1 mL of the receptor was added of 50 μL of 70% *v*/*v* perchloric acid to precipitate albumin, and centrifuged (12,000 rpm, 15 min). IMQ quantification was performed by HPLC-fluorescence. The extraction procedure from epidermis and dermis and the recovery from the receptor solution were previously validated [15]. Permeation and accumulation experiments were also performed using nile red-loaded TO20 micelles (prepared as in Section 2.8.) to visualize the skin distribution of the fluorescent probe by two-photon microscopy.

### 2.10. Statistical Analysis

All data presented in text, figures and tables are reported as mean value ± SD. The significance of the differences between the results was assessed using Student’s *t*-test. Differences were considered statistically significant when *p* < 0.05.

### 2.11. Two-Photon Microscopy Method

Porcine skin samples were analyzed with a two-photon microscope Nikon A1R MP+ Upright equipped with a femtosecond pulsed laser Coherent Chameleon Discovery (~100 fs pulse duration with 80 MHz repetition rate, tunable excitation range 660–1320 nm). A 25× water dipping objective with numerical aperture (NA) 1.1 and 2-mm working distance was employed for focusing the excitation beam and for collecting the two-photon excited fluorescence (TPEF) and the second harmonic generation (SHG) signals. TPEF/SHG signal was directed by a dichroic mirror to a series of four non-descanned detectors (three high sensitivity GaAsP photomultiplier tubes and a multi-alkali detector) allowing fast image acquisition. The four detectors were preceded by optical filters allowing the simultaneous acquisition of four separated channels: blue channel (415–485 nm), green channel (506–593 nm), red channel (604–679 nm) and far-red channel (698–750 nm). Imaging overlay of the four channels and processing was performed by the operation software of the microscope. Additionally, a fifth photomultiplier GaAsP detector, connected to the microscope through an optical fiber and preceded by a dispersive element, was used to record the spectral profile of the TPEF/SHG signal (wavelength range 430 to 650 nm with a bandwidth of 10 nm).

For microscope observations, the full-thickness skin samples were placed in a dedicated plexiglass holder and saline solution was used to dip the objective and to avoid dehydration. The samples were left at room temperature under these conditions for 20 min before measurements, to allow complete thawing. Z stack acquisitions of the porcine skin samples were taken from the stratum corneum to the dermis (150–350 μm deep, with steps between 0.8–1.5 μm depending on the experiment). Images were acquired with a typical field of view of 500 × 500 μm (with 1024 × 1024 pixels definition) and an average acquisition time per image between 2 and 8 s in order to achieve a suitable signal-to-noise ratio.

Different excitation wavelengths were used, between 850 and 1100 nm. Tissue autofluorescence and nile red emission fall in the green and red channel (the latter is much more intense and red-shifted independently of the excitation wavelength). Detector gains for the red and green channels were set at equal values for the collection of images of the same sample, except where explicitly reported. When exciting below 970 nm, the SHG signal falls in the blue channel: the gain of this detector is usually set at higher values compared to red and green channels, in order to better appreciate the SHG signal. No significant photobleaching has been observed in our experiments under the conditions employed to acquire both images and emission spectra.

## 3. Results

TPGS, a water-soluble vitamin E derivative (Table 1), has demonstrated the ability to remarkably increase water-solubility of various hydrophobic compounds as a result of micelles formation in solutions [38,39]. Additionally, some authors also report a permeation enhancing effect of TPGS itself [20,24,40]. Finally, micelles could also promote drug uptake into the skin due to their nanometric size, as reported for retinoic acid [41], hydrocortisone [42] and econazole [43]. In this paper, TPGS-based micelles were evaluated to increase imiquimod solubility and promote its uptake into epidermis and dermis.

### 3.1. Imiquimod Solubilisation

Imiquimod is a small molecule (molecular weight of 240.3 g/mol) with a very low solubility in water (0.6 µg/mL [44]; 2.4 µg/mL [45]). As the drug is a weak base (pka = 7.3), its water-solubility could be slightly improved by protonation at acidic pH [44]. We evaluated the effect of different formulations on imiquimod solubility. The results obtained are summarized in Table 2.

IMQ solubility was firstly evaluated in solutions containing 20 mM TPGS. This concentration is well above the CMC, that was previously measured and resulted approximately 0.1 mM [23]. This polymer previously demonstrated to significantly increase the solubility of hydrophobic compounds such as dexamethasone [46], cyclosporine [23], econazole [47], taxanes [48], minoxidil [40] and simvastatin [49]. Furthermore, TPGS addition to tetramethylguanidinium tetradecanoate micelles was able to increase imiquimod encapsulation efficiency and drug loading [50]. Even though, IMQ solubility in 20 mM TPGS was lower than 20 µg/mL. This disappointing result is probably linked to the low affinity of imiquimod for the hydrophobic micellar core, made of tocopherol, together with the small size of the hydrophobic block (approximately 400 Da), that has been correlated with a low efficiency of encapsulation [51,52,53].

#### 3.1.1. Effect of Co-Encapsulation of Fatty Acids and Oleic Acid Esters

**Figure 1:**
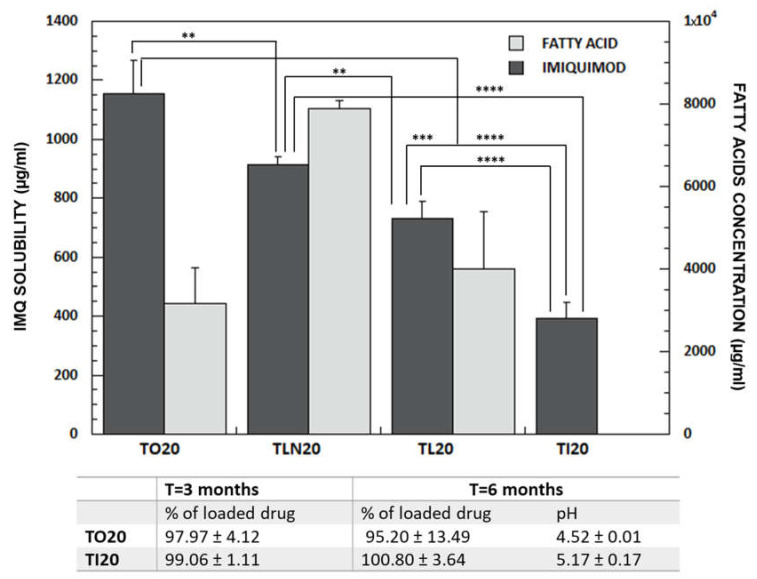
Dark bars indicate IMQ solubility (mean ± SD, Y axis on the left hand) in TPGS 20 mM solutions saturated with oleic (TO20), linolenic (TLN20), linoleic (TL20) and isostearic acid (TI20). Light bars represent the content (mean ± SD, *n* ≥ 3) of fatty acids (Y axis on the right hand). In case of isostearic acid, quantification was not performed due to the absence of a detection method (isostearic acid is not an UV-absorber). The table reports the % of the drug in the formulations after 3 and 6 months from preparation (storage at room temperature) as well as the value of the pH after 6 months. The exact composition of vehicles, with values of drug solubility and fatty acids content is reported in Table 2. (**** *p* < 0.0001, *** *p* < 0.001, ** *p* < 0.01).

In order to increase drug loading, we evaluated the possibility of co-encapsulate a lipophilic compound that could, at least in principle, enter the micellar core and increase its affinity for the drug. This kind of strategy has already been used for enhancing drug encapsulation in other nanocarriers such as niosomes [25] and lipid-based particles [26,27]. As fatty acids have previously demonstrated good imiquimod solubilization properties [15,44], we studied their co-encapsulation into TPGS micelles. Compounds evaluated, liquid at room temperature, were isostearic (C18:0), oleic (C18:1), linoleic (C18:2) and linolenic (C18:3) acids; their structure and relevant physicochemical properties are reported in Table 1. Fatty acids were added to a 20 mM TPGS solution at the saturation and the excess was removed by filtration to obtain a limpid micellar formulation, afterward loaded with IMQ. IMQ solubility values are reported in Table 2 and Figure 1, together with the amount of fatty acid loaded, quantified by HPLC.

The solubility value of IMQ in TPGS/fatty acids mixtures was, in all cases, higher than in TPGS alone, supporting a synergistic solubilizing effect produced by the incorporation of fatty acids in the micellar core: in the absence of TPGS, oleic acid water solubility resulted lower than 60 µg/mL (in agreement with literature data [54]), while in the presence of TPGS, its solubility was higher than 3 mg/mL, testifying its loading in the micelles. Features such as molecular weight, chain length, unsaturation [55], pK_a_ [56] and log P_octanol/water_ can affect fatty acids ability to accumulate in the micellar core and impact on micelles internal structure. For instance, unsaturated fatty acids, in consideration of their ability to reduce lipidic molecules packing in model membranes [57], could also increase the fluidity of micellar hydrophobic core facilitating the incorporation of the drug.

From Figure 1 it is evident that linolenic acid has the highest affinity for micelles core, while oleic acid has the lowest (quantification of isostearic acid was not possible, as it is not UV-absorber). However, the amount of fatty acid loaded was not proportional to the imiquimod solubility. In fact, the highest solubility (1154.08 ± 112.78 µg/mL) was reached with oleic acid, while linolenic, linoleic and isostearic acid gave an IMQ solubility respectively of 912.59 ± 27.50 µg/mL, 731.77 ± 52.25 µg/mL and 393.24 ± 13.34 µg/mL (Figure 1).

It is well known that one of the main problems of micellar formulations is represented by the low stability which is affected by several parameters including the cohesion of the hydrophobic core [53]. In case of TL20 and TLN20, drug precipitation occurred after some weeks. The reason is linked to the expulsion of linolenic and linoleic acid from the TPGS micelles. In fact, fatty acid concentration decreased from 7898.09 ± 180.30 to 280.53 ± 7.99 µg/mL (TLN20) and from 3998.00 ± 140.60 to 258.33 ± 22.25 µg/mL (TL20). In case of TO20 and TI20 micelles, on the contrary, no precipitation occurred and IMQ concentration remained stable up to 6 months from preparation (Figure 1). Additionally, the pH remained substantially stable as well as micelles size (see Section 3.2). The difference in stability between the different fatty acids can be due to their different 3D structure, lipophilicity or pKa (Table 1). Another reason could be linked to the lower chemical stability of linoleic and linolenic acid, caused by the number of unsaturation, which increases their tendency to oxidate.

As the presence of oleic acid demonstrated a positive impact on IMQ solubilization and a good stability over time, we also evaluated the possible solubilizing effect of lipophilic surfactants composed of oleic acid esterified with different polar heads, namely glycerol (Peceol™, formulation TP20), sorbitan (Span^®^ 80, formulation TSP20) or a bigger and more hydrophilic one (Plurol^®^ Oleique, formulation TPO20) (see structures in Table 1). This could help in defining the influence of hydrophilic groups features on IMQ solubility. In all cases, IMQ solubility was below 200 µg/mL (Table 2), suggesting that the presence of a free carboxylic group is essential either for entering the micellar core or for solubilizing the drug. Indeed, from one side the higher MW and hindrance could have prevented the penetration of the hydrophobic chain in the micellar core, and, on the other side, IMQ solubility could be related to an interaction between the positively charged drug (pKa = 7.3) and the fatty acid, with a beneficial influence on IMQ accumulation in the hydrophobic micellar core [58,59].

#### 3.1.2. Effect of TPGS Concentration

**Figure 2:**
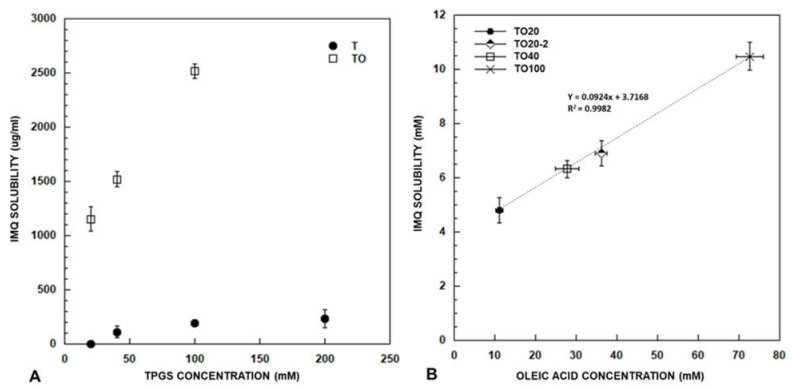
Panel (**A**) represents IMQ solubility (mean ± SD) in TPGS at concentration of 20, 40, 100 e 200 mM with (TO) and without oleic acid (T). Panel (**B**) reports the correlation between oleic acid concentration (mM; mean ± SD) and IMQ solubility (mM; mean ± SD) in micelles with TPGS at concentration of 20 (produced by using both method 1 and 2), 40 and 100 mM (produced by using method 1).

When IMQ was added to 40, 100 and 200 mM TPGS, an increased solubility was obtained (Figure 2 panel A), however very limited and not linearly correlated with TPGS concentration. As oleic acid demonstrated to be the best co-solubilizing agent among all the compounds tested, solutions of TPGS at concentration of 40 and 100 mM were saturated with oleic acid. It was not possible to obtain micellar formulations from 200 mM TPGS saturated with oleic acid, due to the formation of a gel-like system [37] that prevented the separation of the oily phase.

The result (Figure 2, panel A) highlights an increase in solubility roughly proportional to TPGS concentration: in this case TPGS role in drug solubilization appears to be pivotal. By analyzing also oleic acid content, it is clear (Figure 2 panel B) that a linear correlation exists between IMQ solubility and oleic acid concentration, even if the 10-folds increase in oleic acid registered moving from TPGS 20 mM to TPGS 100 mM, reflects in only 2.5 increase in IMQ solubility. The slope of the line indicates that approximately 10 molecules of oleic acid are necessary for the solubilization of 1 molecule of (IMQ), however, the Y-axis intercept suggests that also other phenomena are involved.

#### 3.1.3. Effect of Preparation Method

The important role of oleic acid content on IMQ solubilization is also evident if considering a different method used to saturate the aqueous phase with oleic acid (Section 2.4.2). In this case, the obtained 20 mM TPGS micellar formulation (TO20-2, Figure 2 panel B) loads a higher oleic acid amount and, as a consequence, a higher amount of IMQ (1659.34 ± 109.87 µg/mL). However, the oleic acid is not completely solubilized in micelles TO20-2 as testified by a slight opalescence and by the results of the DLS analysis (see loaded TO20-2 in Table 3).

A further preparation method was also considered (Section 2.4.3). In this case, IMQ was first dissolved at the saturation (approximately 75 mg/mL [15]) in oleic acid and the obtained solution was used to saturate the 20 mM TPGS micelles. This method, evaluated to assess the direct loading of a possible oleic acid-IMQ ion pair, brought to a very low drug solubility (304.76 ± 4.06 µg/mL).

### 3.2. Size Determination

As the size of nano-systems has been reported to play a key role in drug delivery to skin deepest layers, determination of micelles dimension by DLS is considered fundamental to better describe their behavior when applied to skin [17,60].

TPGS micelles size has been previously determined both by DLS and by X-ray scattering [47] and resulted approximately 12 nm. Images from literature [21,61] showed a spherical shape. The results obtained here (T20, T40, T100) are in reasonable agreement with this value (Table 3). Apparently the PDI decreases as the polymer concentration increases. No variation of micelles size was registered before and after loading with the drug and, in case of T40 and T100, the PDI decreased probably due to improved stability and cohesion of micelles caused by the development of hydrophobic interactions between the core and the lipophilic drug incorporated [53]. The addition of oleic acid did not substantially change micelles size.

Multimodal particle size distribution was obtained after addition of surfactants such as Peceol™, Plurol^®^ Oleique and Span^®^ 80 to the 20 mM TPGS solution in case of both blank and IMQ-loaded micelles (Table S3).

The evaluation of micelles size has been used also to further confirm TO20 and TI20 micelles stability over time which demonstrated to have, respectively, dimensions of 14.90 ± 0.13 nm and 13.40 ± 0.16 nm after 3 months from preparation. In case of TO20 (further investigated in the manuscript), zeta potential was also measured. Blank micelles had slightly negative zeta potential (−5.82 ± 0.44 mV), in agreement with pegylated nanocarriers.

### 3.3. Loading of Polymeric Micelles in Hydrogels

Rheological properties of a formulation play a very important role in dermal delivery, as they influence the retention time on skin surface and even impact on patients’ acceptability [62]. In order to preserve the micellar structure, a careful evaluation of the thickening agent should be done. In particular, after incorporation, the behavior of micelles, with specific attention to their diffusion capacity, should be investigated as the nature of polymeric chains as well as their disposition in the hydrogel matrix could prevent micelles release from the system [63]. Polymers for hydrogels preparation were selected considering different characteristics in terms of molecular weight and degree of ionization (neutral or negatively charged polymers). Both natural and synthetic polymers were evaluated. The concentration has been determined empirically on a case-by-case basis, to reach the adequate viscosity for cutaneous application.

**Figure 3:**
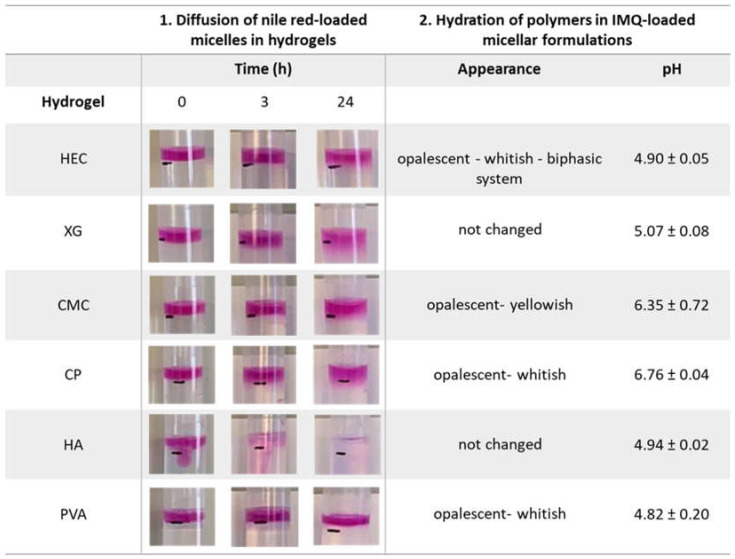
Column 1: diffusion of nile red-loaded micelles in hydrogels at 0, 3 and 24 h from deposition. The black mark on the tube represents the separation surface between the hydrogel and the nile red solution deposed (for a better understanding see Figure S1). Column 2: hydrogels appearance after hydration of polymers in IMQ-loaded TO20 micelles and correspondent pH values (mean ± SD). Hydrogel are indicated as HEC (hydroxyethyl cellulose), XG (xanthan gum), CMC (sodium carboxymethylcellulose), CP (Carbopol^®^), HA (sodium hyaluronate) and PVA (polyvinyl alcohol).

The visual evaluation of the diffusion of nile red-loaded micelles through blank hydrogels has been used as marker of the ability of micelles to be easily released from the formulation (Figure 3). At the deposition, nile red-loaded micelles immediately diffuse in HA hydrogel (see also Figure S1) in which completed diffusional process was observed within few hours. On the contrary, PVA hydrogel was not penetrated by nile red-loaded micelles even after 24 h from deposition. HEC, CMC and CP were characterized by a minimal (if any) micelles diffusion after 24 h. A slightly better result was found for Xanthan gum (see the thickness of the colored layer below the black mark at 24 h in Figure 3, Column 1).

These results were also confirmed when polymers were hydrated in blank and sc-loaded micelles (TO20), Figure 3, Column 2. In fact, in case of HEC, CMC, PVA and CP, the formulation became opalescent or whitish, probably as a result of micelles disruption with consequent release of the oleic acid and the drug. HEC hydrogel showed also a marked phase separation. On the contrary, HA and XG gels preserved their characteristics and were selected to be used for further experiments. Thus, XG and HA hydrogels were identified as the most interesting because of their higher stability and the possibility of micelles diffusion.

Further details on hydrogels aspect after polymers hydration in water and in IMQ-loaded micelles are summarized in Table S2.

### 3.4. Imiquimod Skin Accumulation and Permeation

In order to determine the potentialities of micellar formulations for imiquimod skin delivery, the drug accumulation in full-thickness porcine skin was evaluated after 6 h from the application of different formulations. While IMQ was recovered from epidermis and dermis, no transdermal permeation was observed in the receptor compartment, in which albumin presence ensured sink conditions. The lack of permeation can be attributed to the slow permeation rate and the limited application time (6 h), selected as a reasonable time of formulation persistence on the skin. Formulations tested and accumulation values are listed in Table 4.

#### 3.4.1. Imiquimod Skin Delivery from Micelles

**Figure 4:**
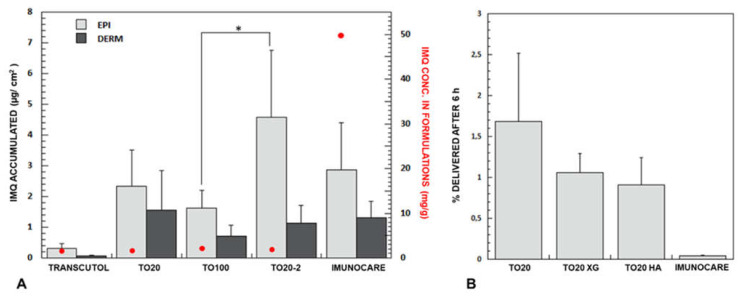
(**A**) IMQ retention (mean ± SD, *n* ≥ 4) in the epidermis and dermis, red dots (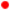) represent IMQ concentration in the formulations applied. Data on Transcutol^®^ saturated solution is from [15]. (**B**) Delivery efficiency (% delivered after 5 h) of TO20 and semisolid formulations. (*p* * < 0.05); *p*-values obtained from *t*-test analysis for epidermis, dermis and total amount of the drug in the skin for all formulations tested are reported in Table S4.

The data obtained in the present work were compared to the one previously obtained starting from a saturated solution of Transcutol^®^ [15]. IMQ concentration in this vehicle is very similar to TO20 micelles (namely 1.11 ± 0.07 µg/mL), it has the same thermodynamic activity (equal to 1, they are both at the saturation) and, being Transcutol^®^ a hydrophilic compound, IMQ should have a similar tendence to partition in the SC. However, as illustrated in Figure 4, drug accumulation into the skin from TO20 micelles is 10-fold higher compared to Transcutol^®^ saturated solution. In particular, the micellar formulation is able to increase penetration depth as dermal levels resulted 25-fold higher for TO20 than for Transcutol^®^ saturated solution.

By increasing TPGS concentration (and thus IMQ solubility) the skin accumulation did not change substantially, and we even found for TO100 a (not-statistically significant) reduction of IMQ skin retention. This could be ascribed to the slightly higher viscosity of the sample, probably due to micelle–micelle interactions [64,65] that limited their freedom of diffusion and thus their interaction with the SC. Additionally, the result supports the substantial absence of penetration enhancing activity of TPGS for imiquimod skin delivery, as recently reported also for rapamycin by Quartier et al. [61].

The formulation TO20-2 gave rise to a significant increase of IMQ accumulation in epidermis, probably attributable to the presence of small oleic acid droplets (Table 4 and Section 3.1.3) and higher IMQ concentration (Table 2). This formulation, however, is not able to promote a deeper IMQ penetration (see dermis concentration), presumably because oleic acid droplets remained trapped in the SC [15].

Based on these results, we can hypothesize that the presence of small micelles (approximately 15 nm in diameter) free to diffuse can significantly enhance IMQ penetration into the skin. The good performance of nanometric micelles was also reported by other authors [16,66,67,68] and was ascribed to the improved accumulation of the nanosystems in the hair follicles as well as to the affinity of the polymers used for both the stratum corneum and the cellular membrane, which results in a strong change in the membranes fluidity, thus promoting the passive uptake.

**Figure 5:**
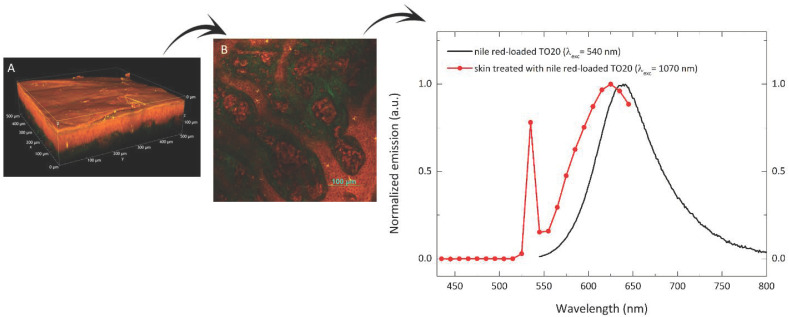
Porcine skin treated with nile red-loaded TPGS micelles. (**A**) Volume rendering of flat porcine skin reconstructed from the Z-stack (Z-step: 0.8 μm, total depth: 151 μm); (**B**) intermediate region between epidermis and dermis, acquired about 80 μm from the sample surface; dermis papillae are evident (in green the collagen) together with annexial, vascular and corpuscular elements; (**C**) comparison between the normalized emission spectrum of an aqueous suspension of nile red-loaded TO micelles (black line) and the emission spectrum acquired in the skin region shown in (**B**) (red line). The sharp peaks at 535 nm (red line) is relevant to the SHG signal. All the images have been acquired with excitation wavelength of 1070 nm. A more complete and detailed view of the flat porcine skin 3D reconstruction is available in Supplementary Movie S1.

In order to visualize the behavior of this formulation on skin tissue, TO20 micelles loaded with nile red were prepared and applied for 6 h on full-thickness porcine skin. As a reference, a blank experiment was also done, by using saline solution as donor. Two-photon microscopy demonstrated to be a very powerful tool for the study of skin structure and fluorescent probe distribution: details on the technique and selection of the optimal excitation wavelength are presented in Appendix A. Figure 5 illustrates the volume rendering of a porcine skin sample where the different skin layers are clearly visible as well as nile red accumulation in SC and viable epidermis; the green signal is referred to the collagen fibers present in the dermis (SHG signal). A more complete and detailed view is available in Supplementary Movie S1: the probe clearly marks all the cellular elements of the skin, i.e., epidermis and also vascular and corpuscular elements in the dermis. From this sample, at an 80 µm depth (Figure 5B), the spectral profile of the emitted/generated signal was also recorded and compared with the emission spectrum collected from nile red-loaded TO20 micelles (Figure 5C) with an Edinburgh FLS-1000 fluorimeter. Emission of nile red is strongly sensitive to the polarity of the environment [69]. The emission of nile red in porcine skin is shifted towards shorter wavelengths compared to the emission of nile red in TO20 micelles. The shift of the emission spectrum of nile red indicates a change of environment polarity, suggesting that the fluorescent probe has been released from the micelles. Overall, these images support the capability of TO20 micelles to efficiently deliver the probe to the skin, integrating the data obtained with imiquimod.

**Figure 6:**
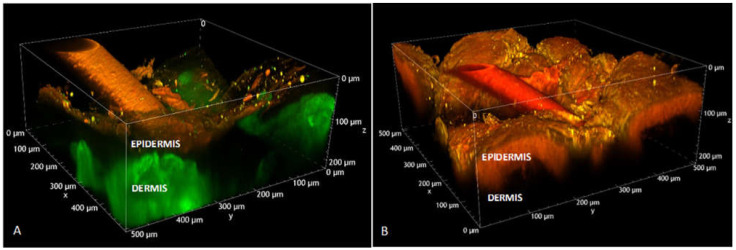
Volume renderings of porcine hair follicles reconstructed from Z-stacks acquired for excitation wavelength of 1100 nm. (**A**) skin treated with saline solution (Z-step: 1.5 μm, total depth: 222 μm); laser power: 102.4 mW. (**B**) Skin treated with nile red-loaded TO20 (Z-step: 1 μm, total depth: 222 μm); laser power: 19.2 mW; the detector gain and the laser power were lower for (**B**) compared to (**A**) to avoid saturation; for this reason, the SHG of dermis collagen is not visible in panel (**B**).

**Figure A3:**
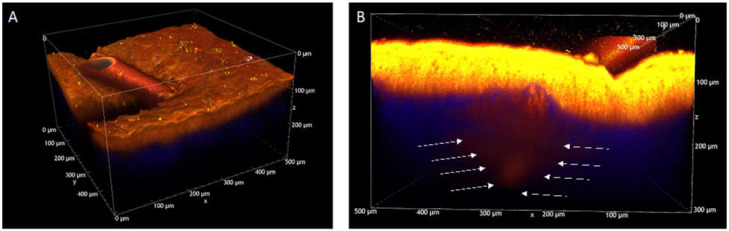
Volume rendering of a porcine hair follicle reconstructed from the Z-stack (Z-step: 0.8 μm, total depth: 307 μm) acquired with excitation wavelength of 850 nm. (**A**) 3D rendering overview; (**B**) XZ view with red channel saturated using LUTs, in order to highlight the red signal in the innermost layers of the tissue (arrows). An animated view of the hair follicle porcine skin 3D reconstruction is available in Supplementary Movie S3.

A specific skin section with a hair follicle, presented in Figure 6, was analyzed and compared to a control sample (skin treated with saline solution). In this case nile red accumulation in the hair shaft and its penetration into the hair follicle were demonstrated (see also Figure A3). Details on the technique used and further images are presented in Appendix A.

#### 3.4.2. Imiquimod Skin Delivery from Semisolid Formulations

When micelles were included in a gel, essential for skin application [61], drug delivery was slightly reduced, in particular to the dermis (Table 4). However, this reduction is not statistically significant, and the result obtained support the selection of XG and HA as thickening agents capable to preserve micelles diffusion. A similar result was obtained by Lapteva et al. [70] who used mPEG-hexPLA micelles embedded in a carboxymethyl cellulose gel.

Finally, the results can be compared with the commercial cream. The drug level found in epidermis and dermis are comparable to the formulation prepared in the present work, however, the delivery efficiency of Imunocare^®^ (IMQ concentration: 50 mg/g) is extremely low (Figure 4, panel B), and 42-fold lower than TO20. Thus, the micellar formulation is able to produce similar epidermis and dermis accumulation with a 40-fold lower concentration, with possible advantages in the therapeutic outcome and reduced systemic side effects in case of damaged skin. These side effects, although rare, are fever, vertigo, myalgia and, anecdotal reports of distant inflammatory mucosal reactions [71]. Additionally, IMQ is characterized by important local side effects. Some of them (irritation and erythema) are an extension of the pharmacologic effect, while others (skin infection, skin hypo and hyper pigmentation, alopecia) are unrelated. The dramatic reduction of the concentration of the vehicle could in principle have an effect also on some of these manifestations. Additionally, the presence of TPGS, that can deliver vitamin E to the epidermis, can mitigate some local reactions, as indicated in a recent paper [72]. Indeed, TPGS can be hydrolyzed to vitamin E [23], thanks to the presence of esterase activity in the stratum corneum.

## 4. Conclusions

In the present paper, TPGS-based micelles co-loaded with oleic acid were prepared, characterized and exploited for solubilizing and delivering imiquimod to the skin. A linear correlation was found between imiquimod solubility and oleic acid amount in the micelles, underlining the relevant role of this fatty acid in imiquimod formulation. These micelles, prepared by a very easy procedure, were stable at least for 6 months and, applied ex vivo to porcine skin, gave rise to a relevant imiquimod skin deposition, with a delivery efficiency 40-fold higher than the commercial cream. A careful selection of the thickening agents permitted to obtain a gelified formulation preserving the skin delivery performance of the micelles.

In consideration of these findings, it is possible to affirm that the formulation TO20 is a promising vehicle for imiquimod delivery to the skin and, hopefully, for reducing side effects, due to the lower drug concentration in the vehicle and the presence of TPGS.

## Figures and Tables

**Table 1:** Drug and excipients used for micelles preparation with their main features and chemical structures.

#	Compound	Log P [29]	HLB	pKa	H-Bond Capacity [30] (Donor-Acceptor)	Molecular Weight (g/mol)
**1**	Imiquimod	2.9	-	7.3 [29]	2–4	240.30
**2**	TPGS	-	13.2 [31]	-	1–6	1513.00
**3**	Oleic acid (18:1)	7.19	-	4.90 [29]–9.85 [32]	1–2	282.50
**4**	Linolenic acid (18:3)	5.95	-	4.90 [29]–8.28 [32]	1–2	278.43
**5**	Linoleic acid (18:2)	6.65	-	4.78 [29]–9.24 [32]	1–2	280.45
**6**	Isostearic acid (18:0)	7.2	-	4.78 [29]	1–2	284.50
**7**	Peceol™	-	3 [33]	13.16 [34]	2–4	356.54
**8**	Plurol^®^ Oleique CC497	-	3 [35]	-	20–27	1023.20
**9**	Span^®^ 80	-	4.3 [36]	-	3–6	428.60
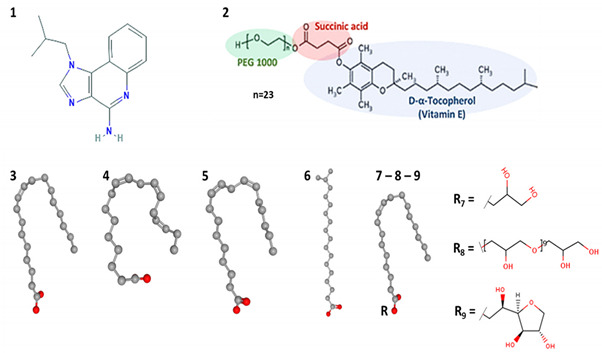						

**Table 2:** Composition of micellar solutions with their method of preparation and the correspondent code. Drug solubility, concentration of the co-solubilizing agent and pH of the formulations are also reported. Data are reported as mean ± SD, *n* ≥ 3.

Code	Composition	Method	Imiquimod Solubility (µg/mL)	Fatty Acid Content (µg/mL)	pH
T20	TPGS 20 mM	1	1.70 ± 0.63	-	6.14 ± 0.14
T40	TPGS 40 mM	1	109.81 ± 53.77	-	5.06 ± 0.45
T100	TPGS 100 mM	1	188.76 ± 6.76	-	5.37 ± 0.43
T200	TPGS 200 mM	1	231.51 ± 84.21	-	-
TO20	TPGS 20 mM + oleic acid	1	1154.08 ± 112.78	3156.69 ± 882.31	4.79 ± 0.03
TO40	TPGS 40 mM + oleic acid	1	1520.61 ± 72.98	7871.15 ± 808.39	4.69 ± 0.02
TO100	TPGS 100 mM + oleic acid	1	2517.56 ± 70.26	20519 ± 956.79	4.92 ± 0.03
TLN20	TPGS 20 mM + linolenic acid	1	912.59 ± 27.50	7898.09 ± 180.30	4.76 ± 0.06
TL20	TPGS 20 mM + linoleic acid	1	731.77 ± 52.25	3998.00 ± 140.60	5.09 ± 0.04
TI20	TPGS 20 mM + isostearic acid	1	393.24 ± 13.34	n.d. ^a^	5.34 ± 0.01
TP20	TPGS 20 mM + Peceol™	1	180.21 ± 35.99	-	5.30 ± 0.10
TPO20	TPGS 20 mM + Plurol^®^ Oleique	1	126.55 ± 5.52	-	5.99 ± 1.00
TSP20	TPGS 20 mM + Span^®^ 80	1	115.34 ± 76.82	-	6.20 ± 0.13
TO20-2	TPGS 20 mM + oleic acid	2	1659.34 ± 109.87	8683.54 ± 344.36	4.63 ± 0.03
TO20-3	TPGS 20 mM + oleic acid	3	304.76 ± 4.06	n.d.	3.44 ± 0.01

n.d. not determined; ^a^ not UV-absorber.

**Table 3:** Size (mean ± SD), intensity and PDI of blank and IMQ-loaded micelles at 0 days from preparation. Micelles were diluted 1:10 with high purity water prior to analysis.

Formulation	Blank Size (nm)	Intensity (%)	PDI	Loaded Size (nm)	Intensity (%)	PDI
T20	14.43 ± 0.39 647.80 ±37.75	86.93 26.13	0.28	14.01 ± 0.68 845.56 ± 289.37	86.33 12.77	0.29
T40	13.71 ± 0.26	95.03	0.20	12.66 ± 0.11	100	0.08
T100	12.26 ± 0.22	100	0.07	11.95 ± 0.06	100	0.06
TO20	16.02 ± 0.61	96.07	0.17	15.31 ± 0.69 230.66 ± 66.18	61.67 42.96	0.57
TO20-2	40.48 ± 0.52	100	0.12	16.74 ± 0.62 222.77 ± 2.08	13.33 86.67	0.63
TO40	30.40 ± 1.19	99.43	0.22	20.13 ± 0.48	100	0.25
TO100	13.42 ± 0.11	93.93	0.21	12.63 ± 0.06	100	0.04
TI20	14.69 ± 0.18 511.77 ± 64.94	85.85 23.23	0.238	13.57 ± 0.76 612.44 ± 383.32	79.55 17.64	0.36

**Table 4:** Amount of IMQ accumulated (mean ± SD, *n* ≥ 4) from formulations applied for 6 h in infinite dose conditions.

Formulation	Epidermis (µg/cm^2^)	Dermis (µg/cm^2^)	Total (µg/cm^2^)
*TRANSCUTOL^®^ SS* [15]	0.32 ± 0.14	0.06 ± 0.04	0.38 ± 0.17
TO20	2.32 ± 1.18	1.56 ± 1.28	3.89 ± 1.93
TO100	1.63 ± 0.57	0.71 ± 0.34	2.34 ± 0.53
TO20-2	4.57 ± 2.19	1.14 ± 0.56	5.46 ± 2.56
Imunocare^®^	2.87 ± 1.51	1.32 ± 0.53	4.19 ± 0.99
TO20 XG	1.64 ± 0.65	0.80 ± 0.24	2.44 ± 0.54
TO20 HA	1.59 ± 0.65	0.50 ± 0.22	2.10 ± 0.77

**Figure A1:**
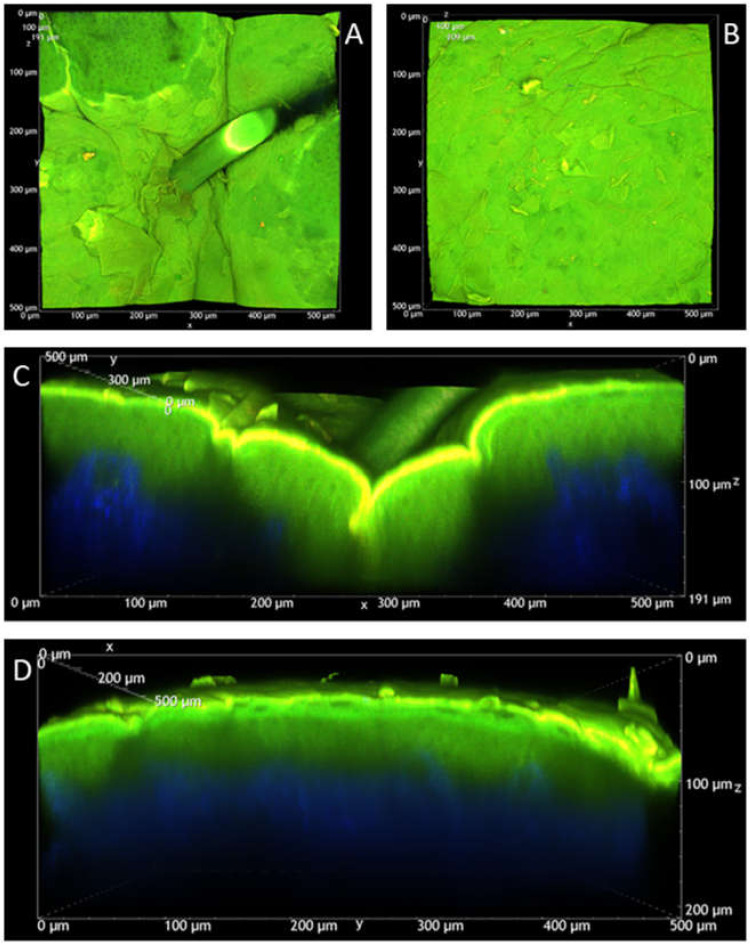
Volume renderings of untreated porcine skin samples reconstructed from the Z-stacks (Z-step: 1 μm) acquired with excitation wavelength of 850 nm. XY (**A**) and XZ (**C**) views of a hair follicle (total depth: 191 μm); XY (**B**) and YZ (**D**) views of a flat skin region (total depth: 209 μm). A more complete and detailed view of the flat skin region 3D rendering is available in Supplementary Movie S2. The blue signal is due to the second harmonic generation (SHG) of the collagen present in the dermis. The green signal is due to SC and epidermis autofluorescence when irradiated at 850 nm.

**Figure A2:**
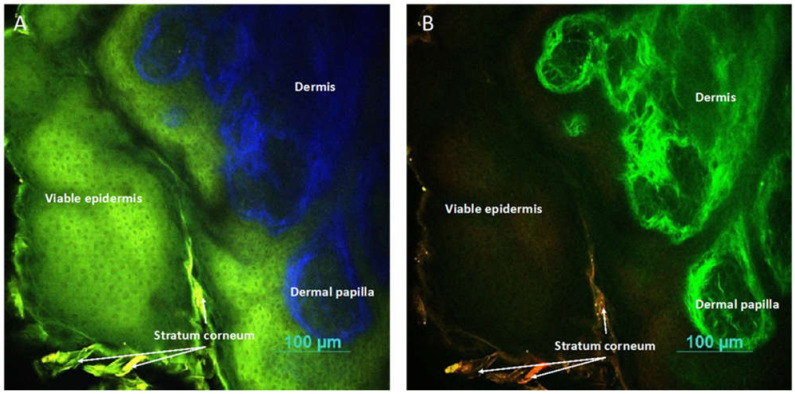
Untreated porcine skin, autofluorescence and SHG images of non-flat intermediate regions between epidermis and dermis. The images have been captured about 80 μm under the surface, with two different excitation wavelengths: 900 nm (**A**) and 1100 nm (**B**). In (**A**) SHG falls in the blue channel, while in (**B**) SHG falls in the green channel.

**Figure A4:**
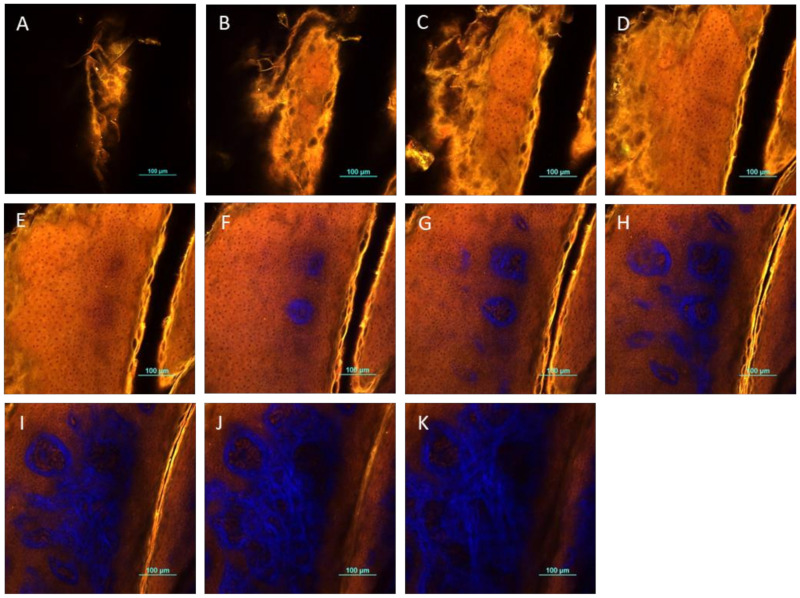
TPEF/SHG images of different layers of porcine skin treated with nile red-loaded TPGS micelles. Starting from the surface, each image was acquired 10 μm deeper than the previous one, increasing depth from (**A**–**K**). All the images have been captured using 850 nm as excitation wavelength.

## Data Availability

The data presented in this study are available on request from the corresponding author.

## Supplementary Materials

The following are available online at https://www.mdpi.com/article/10.3390/pharmaceutics13091476/s1, Table S1: Range of calibration curves, RSD% (relative standard deviation %) and ER% (relative error %) of the fatty acid considered; Table S2: Hydrogels composition, with their codification, appearance and pH values. Table S3: Size (mean ± SD), intensity and PDI of blank and IMQ-loaded micelles of TP20, TPS20 and TPO20. Table S4: p values obtained from t-test analysis for epidermis, dermis and total amount of the drug in the skin for all formulations tested; Figure S1: Hydrogels before and after deposition of 200 µL of Nile-Red loaded micellar solution. Supplementary Movie S1: 3D reconstruction of porcine skin after 6 h treatment with nile-red loaded TO20 micelles (excitation wavelength: 1070 nm); Supplementary Movie S2: 3D reconstruction of untreated porcine skin (excitation wavelength: 850 nm); Supplementary Movie S3: 3D reconstruction of hair follicle after 6 h treatment with nile-red loaded TO20 micelles (excitation wavelength: 850 nm).

## Appendix A

Two-photon microscopy images of blank skin samples and selection of the excitation wavelength.

Figure A1 reports volume renderings of untreated porcine skin samples reconstructed from the Z-stacks when excited at 850 nm. In this condition, the dermis is blue, due to the second harmonic generation (SHG) signal typical of collagen fibers [73], while stratum corneum and viable epidermis are characterized by a marked green autofluorescence. The same appearance has been detected for control samples treated with saline solution and blank TO20 formulation.

Longer excitation wavelengths were then evaluated: Figure A2 refers to a skin sample (80 µm deep) where, due to the inclination of the tissue, SC, viable epidermis and dermis are visible on the same plane. The two panels refer to excitation at 900 nm (panel A) and 1100 nm (panel B). From the image it is evident that at 900 nm, green autofluorescence is still present in SC and epidermis while, when the tissue is excited at 1100 nm, autofluorescence is not present in case of viable epidermis and only a very limited red signal is present on the SC. This last excitation wavelength was then used to obtain the images reported in Figure 5 and Figure 6 of the main text.

Two-photon microscopy images (excitation at 850 nm) of skin samples treated with nile red-loaded TO20 micelles

Despite the spectral overlap between tissue autofluorescence and nile red emission, the latter is much more intense and also images collected with excitation at 850 nm can help in describing nile red penetration into the skin from TO20 micelles. Figure A3 confirms the data previously shown (see Figure 5 and Figure 6) regarding nile red retention in stratum corneum and epidermis. Panel B of the same figure, obtained with red channel saturated using LUTs, highlights the deep penetration of the probe in the hair follicle.

Figure A4, represents a sample of micelles-treated porcine skin at different depths, where images are collected exciting the sample at 850 nm. It is possible to visualize the presence of skin furrows whose width is reduced by proceeding towards the deeper layers and nile red accumulation in the epithelial cells. Furthermore, at 50 µm depth, collagen fibers from the dermis become clearly visible. The circular structure of these fibers has to be referred to the dermal papillae, ridge-like structures extended on the surface area of the dermal-junction in the papillary dermis.
