# Supplementary material for: Improvement of Imiquimod Solubilization and Skin Retention via TPGS Micelles: Exploiting the Co-Solubilizing Effect of Oleic Acid
Source: Pharmaceutics. 2021 Sep 15;13(9):1476. doi: 10.3390/pharmaceutics13091476 (PMC8469695; doi:10.3390/pharmaceutics13091476)
Supplement: Supplementary file 1 [file pharmaceutics-13-01476-s001.zip › pharmaceutics-1375523-SI/Supplementary_materials_and_movies/pharmaceutics-1375523-SI.pdf]

pharmaceutics

# Supplementary Materials: Improvement of Imiquimod Solubilization and Skin Retention via TPGS Micelles: Exploiting the Co-Solubilizing Effect of Oleic Acid

**Martina Ghezzi, Silvia Pescina, Andrea Delledonne, Ilaria Ferraboschi, Cristina Sissa, Francesca Terenziani, De Freitas Rosa Remiro Paula, Santi Patrizia and Nicoli Sara**

**Table S1.** Range of calibration curves, RSD% (relative standard deviation %) and ER% (relative error %) of the fatty acids considered.

| Fatty Acid | Concentrations Interval (μg/ml) | RSD% | RE% | LOQ | | |
|---|---|---|---|---|---|---|
| | | | | LOQ (μg/ml) | RSD% | RE% |
| Oleic acid | 60–600 | < 0.88 | < 2.37 | 60 | 7.33 | 2.58 |
| Linoleic acid | 3–100 | < 2.10 | < 2 | 3 | 6.6 | 7 |
| Linolenic acid | 5–100 | < 5.6 | < 5.6 | 5 | 2.31 | 21 |

**Table S2.** Hydrogels composition, with their codification, appearance and pH values. The appearance of hydrogels was evaluated after hydration of polymers in water, blank TO20 micelles and IMQ-loaded TO20 micelles. In table only the aspect of water-based and IMQ-loaded TO20-based hydrogels is reported as no difference was observed between hydrogels hydrated in blank TO20 and IMQ-loaded TO20 micelles.

| Polymer | Composition | Code | Appearance | | pH | |
|---|---|---|---|---|---|---|
| | | | Water-Based Hydrogels | IMQ-Loaded TO20-Based Hydrogels | Water-Based Hydrogels | IMQ-Loaded TO20-Based Hydrogels |
| Hydroxyethyl-cellulose | 2% | HEC | transparent | opalescent- whitish – biphasic system | 6.75 ± 0.01 | 4.90 ± 0.05 |
| Xanthan Gum | 1% | XG | slightly opalescent | slightly opalescent | 5.92 ± 0.68 | 5.07 ± 0.08 |
| Sodium Carboxymethylcellulose | 4% | CMC | transparent/yellowish | opalescent- yellowish | 6.66 ± 0.09 | 6.35 ± 0.72 |
| Carbopol 934 | 0.5% | CP | transparent | opalescent- whitish | 5.71 ± 0.07 | 6.76 ± 0.04 |
| Sodium Hyaluronate | 1% | HA | transparent | transparent | 6.72 ± 0.03 | 4.94 ± 0.02 |
| Polyvinyl Alcohol | 15% | PVA | transparent | opalescent- whitish | 6.16 ± 0.43 | 4.82 ± 0.20 |

**Table S3.** Size (mean ± SD), intensity and PDI of blank and IMQ-loaded micelles of TP20, TPS20 and TPO20 at 0 days from preparation. Micelles were diluted 1:10 with distilled water prior to analysis.

| | Blank | | | Loaded | | |
|---|---|---|---|---|---|---|
| Formulation | Size (nm) | Intensity (%) | PDI | Size (nm) | Intensity (%) | PDI |
| TP20 | 12.61 ± 0.34<br>265.50 ± 24.68 | 72.53<br>25.57 | 1 | 12.13 ± 0.08<br>172.67 ± 2.75 | 68.63<br>31.37 | 0.98 |
| TPS20 | 14.71 ± 0.26<br>165.83 ± 19.72 | 41.97<br>56.20 | 0.61 | 65.85 ± 37.82<br>122.70 ± 0.00 | 82.53<br>48.9 | 0.49 |
| TPO20 | 13.37 ± 0.36<br>175.17 ± 2.05 | 12.97<br>87.30 | 0.59 | 13.07 ± 0.78<br>249.17 ± 9.77 | 12.30<br>87.73 | 0.65 |

**Table S4.** P values obtained from T-test analysis for epidermis, dermis and total amount of the drug in the skin for all formulations tested.

| | TO20 | TO100 | TO20-2 | TO20 XG | TO20 HA | IMUNOCARE |
|---|---|---|---|---|---|---|
| **TO20** | - | 0.32; 0.24; 0.16 | 0.08; 0.52; 0.20 | 0.29; 0.24; 0.14 | 0.26; 0.10; 0.09 | 0.62; 0.81; 0.84 |
| **TO100** | 0.32; 0.24; 0.16 | - | **0.04**; 0.23; **0.02** | 0.98; 0.67; 0.79 | 0.92; 0.33; 0.61 | 0.19; 0.16; **0.03** |
| **TO20-2** | 0.08; 0.52; 0.20 | **0.04**; 0.23; **0.02** | - | **0.02**; 0.24; **0.01** | **0.02**; 0.05; **0.01** | 0.37; 0.72; 0.41 |
| **TO20 XG** | 0.29; 0.24; 0.14 | 0.97; 0.67; 0.77 | **0.02**; 0.24; **0.01** | - | **0.90**; 0.08; 0.43 | 0.16; 0.11; **0.02** |
| **TO20 HA** | 0.26; 0.10; 0.09 | 0.92; 0.33; 0.61 | **0.02**; 0.05; **0.01** | 0.90; 0.08; 0.43 | - | 0.14; **0.02**; **0.03** |
| **IMUNOCARE** | 0.62; 0.81; 0.84 | 0.19; 0.16; **0.03** | 0.37; 0.72; 0.41 | **0.16**; 0.11; **0.02** | 0.14; **0.02**; **0.03** | - |

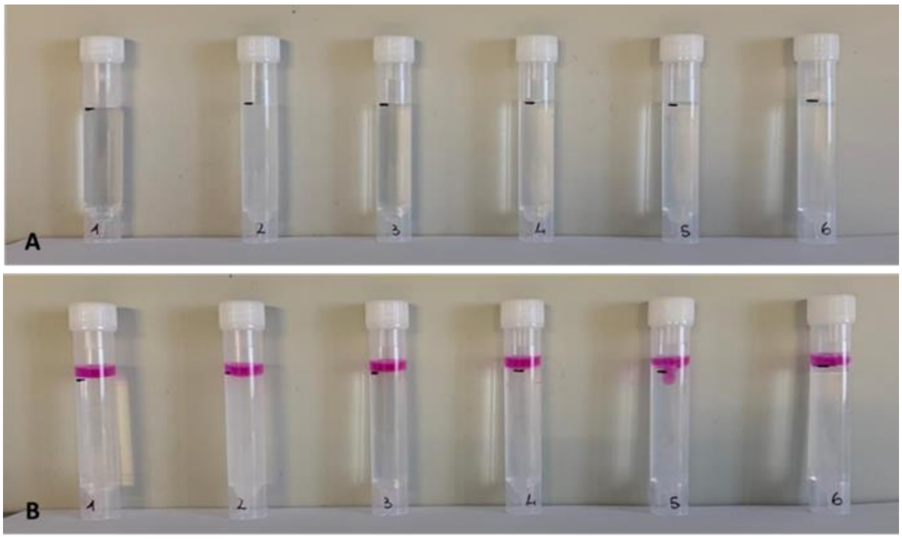


**Figure S1.** Hydrogels of HEC (1), XG (2), CMC (3), CP (4), HA (5) and PVA (6) before (**A**) and after (**B**) deposition of 200 µl of Nile-Red loaded micellar solution.
